# Cases in Private Practice

**Published:** 1871-02

**Authors:** Jno. E. Owens

**Affiliations:** Surgeon St. Luke’s Hospital, Chicago


Article II.—*Cases in Private Practice.* By Jno. E. Owens, M.D., Surgeon St. Luke's Hospital, Chicago.

[Continued from page 20.]

*Remarks.* Besides the drastic cathartic and injection, the treatment, in the first case, consisted of Calomel and Opium in combination, Sweet Spirits of Nitre, and Opium, or Morphia pro re nata. Every effort was made to apply leeches, but in vain. In lieu of the latter, a blister was ordered over the tender part, but this produced anything but a satisfactory result. Fomentations were used from the first. In this case I was in the dilemma of having started with an incorrect diagnosis. Indeed, I know of no class of diseases so obscure, in a diagnostic point of view, whether connected with the alimentary canal in general, with the cæcum and appendix in particular, or with other organs in this locality, as tumors, inflammatory or otherwise, of the abdominal cavity. As the case progressed, however, it became the more obscure, as the prominent symptoms of intussusception were forced more and more in the background. Since this case was under my care, I have learned that every minute circumstance is important, and must be considered, before we can arrive at a correct diagnosis. Localized pain, tenderness, nausea, vomiting, swelling, distension of the abdomen, constipation, and a scanty secretion of urine, characterized the first case. We may have precisely the same chain of symptoms in inflammatory affections of the cæcum and its appendix, whether the inflammation commence in the areolar tissue around the cæcum, in the cæcum itself, in its peritoneal covering, or in the appendix. We may have the same chain, in intestinal obstruction and strangulation, whether the cause be the presence of foreign bodies, impacted feces, gall-stones, strictures of the gut, displacements, brands, adhesions, or gaps in the ementum. These, then, being prominent points of similitude, let us see what points of contrast there are between intussusception or obstruction, inflammation of the cæcum and appendix, from the presence of foreign bodies in the appendix, and simple inflammation of the same. The most frequent form of intestinal obstruction is intussusception. There is a sudden pain, which recurs in paroxysms, is accompanied by constipation, and is usually localized by the patient. At the seat of pain, a firm, dull, resistant tumor, or mass, is usually felt. Nausea, vomiting, prostration, fever, peritonitis, and distention of the abdomen, supervene. The vomiting sooner or later becomes stercoraceous—unless the disease be seated in the upper part of the tube—in some part of the jejunum, for instance, in which case the vomiting will remain bilious for the most part. If the tumor is located about the iliac fossa, fecal vomiting will soon occur. We can well understand how insuperable must be the constipation of a perfectly closed bowel. Sometimes, however, the constipation is not absolute, owing to the constricted part remaining pervious, the liquid contents passing through the intussuscepted part and occasioning a deceptive diarrhœa. There may be, likewise, this same deceptive diarrhœa, should the obstruction be located in the upper part of the tube, and occasionally when it is seated in the large bowel. Indeed, after a time there is likely to be a discharge of blood, serum and mucus, accompanied with considerable tenesmus. These, together with the paroxysmal character of the pain, and the movements of the excited intestines, which may be seen and felt soon after the commencement of the affection, are symptoms that materially assist us in a diagnosis of intussusception. Finally, the symptoms of obstruction force those of inflammation more and more in the background.

The most common affection of the cæcum and appendix, is inflammation. It arises from a variety of causes. When an inflammatory affection of this part of the canal is diagnosed, it is seldom that we can go further, except to remember that it is generally caused by the presence of foreign bodies. Of course. the symptoms vary according to the severity of the inflammation, In forming the diagnosis of inflammation from the presence of a foreign body in the appendix, we must eliminate cancer, psoas-abscess, inflammation of the ovary, abscess of the kidney, abscess in the abdominal walls, cæcal distension, and intussusception. This may done, for the most part, by a critical consideration of the history of the case. Cancer, psoas-abscess, and abscess of the kidney, are chronic affections. In suspected renal abscess, an examination of the urine will negative or confirm the diagnosis. In cæcal distention and inflammation, unless a foreign body be present, the symptoms seldom become so urgent, and are scarcely ever fatal. Ovarian disease usually comes on with disordered menstruation, and the pain is usually lower down in the hypogastrium. The symptoms of abscess in the abdominal parietes are markedly less severe, and the disease, in a very short time, manifests its local character. In the first case—one of the presence of a foreign body in the appendix—the patient was attacked, eight or ten hours after eating chestnuts, with colicy pains in the right iliac-fossa, and was obliged to return from his evening walk. The bowels were somewhat constipated on Friday, 7th. The second day, the pains grew intense; there were constipation, fever, some nausea and vomiting. In the right iliac-fossa an irregular, exquisitely painful tumor, dull on percussion, was detected. There was surrounding tympanitis. The vomited matter was bilious. An invagination at the site of the tumor would, very soon, have been attended with fecal vomiting. The constipation was not insuperable, since there was an evacuation the first part of the third day; four others, containing a little mucus, towards the latter part of the third day, and one on the fourth. None of them contained blood, nor were they characterized by the normal amount of fecal odor. The symptoms of peritonitis rapidly became prominent; the abdomen grew more and more tympanitic; vomiting, with absence of fecal smell, continued; tenderness became more extended; the urine scanty; temporary suppression supervened the third day; the patient, growing more restless, died at the end of six and a half days. This is the history of inflammation of the cæcum and appendix, with perforation of the latter, from the presence of foreign bodies, such as raisin or grape seeds, cherry stones, chestnuts, pins, nails, shot, bristle of a tooth-brush, etc. We may include gall-stones, and fecal and mucous concretions. Cases of perforation generally occur in early life. Habershon says that the detection of foreign bodies in the appendix, without any severe irritation being felt, is by no means uncommon. A pin has been found with its head downwards. Again, he has observed an iron nail, shot, and, if I remember correctly, a mucous or fecal concretion, whose nucleus was the bristle of a tooth-brush, thus lodged, without injury. Inflammation of the appendix and cæcum, from the impaction of foreign bodies, rapidly progresses from bad to worse, especially in cases of ulceration and perforation. Cases like these, commencing with great tenderness and fever, are inflammatory from the first.

The differential diagnosis of simple inflammation of the cæcum and appendix, and that arising from the presence of foreign bodies, is a most difficult subject. Proof of the ingestion of foreign bodies, such as I have enumerated, and the greater prominence and obstinacy of the symptoms of the case look more toward the latter. If gall-stones have previously been detected in the stools, or should previous symptoms have pointed to their passage from the gall-ducts, or gall-bladder, the probability of their presence in the appendix or cæcum must be considered.

From the *post-mortem* consideration of the first case, I need scarcely say how wrong was the exhibition of drastic cathartics, which not only do no good, but positive harm, by increasing the frequency of vomiting, and aggravating the other symptoms. Unless the case be one of distension of the cæcum, from feces and gas, active cathartics are contra-indicated. In the matter of diagnosis, something may be gained by the administration of a mild aperient, but should we find that the constipation does not yield, it is erroneous to persist in this direction. Leeches, fomentations, rest, and chloral-hydrate, are the remedies to be employed. Morphia and opium are likely to add to the nausea and vomiting; hence chloral-hydrate is preferable. In the second case of simple inflammation under this treatment, the patient made a good recovery.

